# Revamping the Perished: The Management of Internal Tooth Resorption

**DOI:** 10.7759/cureus.61214

**Published:** 2024-05-28

**Authors:** Manoj Chandak, Swayangprabha Sarangi, Payal Chaudhari, Abhilasha Dass

**Affiliations:** 1 Department of Conservative Dentistry and Endodontics, Sharad Pawar Dental College and Hospital, Datta Meghe Institute of Higher Education and Research, Wardha, IND

**Keywords:** endodontics, mta, gutta percha, thermoplasticised, ododontoclast, internal resorption

## Abstract

Root resorption is a challenging endodontic case in terms of the management of both hard and soft tissues in patients. It requires thorough knowledge, the proper choice of material, and regular follow-ups. Several etiological factors are responsible for the susceptibility of the tooth to resorption. The most common are dental caries and trauma. This case report sheds light on the etiopathogenesis of the development of internal root resorption and the clinical management of the resorptive defect. It also focuses on the need for proper diagnostic methodology for treating such complex defects.

## Introduction

The phenomenon of root resorption is an undesirable and uneventful pathological process that occurs due to the mutilation of hard tissues due to regressive odontoclastogenic action occurring in the anatomic apex of the tooth. The incidence of this occurs more in permanent tooth roots than in primary ones.

The classification of root resorption is broadly based on its location, i.e., the type of resorption that occurs on the innermost aspect (adjoining the pulpal chamber) is the internal root resorption, whereas the type of resorption occurring on the outer periphery of the root canal is the external type of root resorption [1]. A variety of resorptive phagocytic cells, described as odontoclasts, surround the spaces around which resorption takes place. These cells are responsible for the degeneration that takes place through the release of acidic enzymes [2].

Internal root resorption, known for its advanced and deteriorating type of destruction, occurs mainly along the walls of the root canals, particularly in the middle and apical parts. It involves the intraradicular dentin and dentinal tubules as a consequence of clastogenic activity induced by the phagocytic cells [3].

The type of internal root resorption taking place in the radicular portion as a result of inflammatory processes due to enhanced destruction of dentin surrounding the tubules mostly occurs within the confines of the canal walls. This clastic activity leads to the filling up of the resorptive bays and lacunae with a substance that occurs visibly similar to granulation tissue or like a mixture of bone and cementum-like mineralized tissue [4].

The etiological factors for the two types of resorption are different in that internal resorption has an inflammatory nature of pathogenesis in comparison to external resorption. The protection of the tooth occurs due to the coat of cementum present on the tooth surface, which protects the external surface from resorption. Additionally, a layer of predentin is also present to restrict the spread of clastic processes on the internal dentin [3]. The procedure of diagnosing such resorptive defects is tedious, and due to limited available resources and a lack of advanced radiographic techniques, the procedure sometimes becomes difficult to treat, even though it might seem effortless. The major restraint of such procedures is that a two-dimensional image provides restricted information, whereas the need is for a structured three-dimensional image. The use of conventional radiographs does not help in giving a three-dimensional overview of the resorptive defects [5,6]. However, CBCT addresses this limitation thus helping to arrive at an accurate diagnosis.

This case report focuses on the use of CBCT in the diagnosis and management of internal root resorption in a maxillary central and lateral incisor.

Internal inflammatory root resorption is a rare occurrence, and its etiology and pathology are not completely understood. The exposure or iatrogenic damage of the predentin, organic sheath, and odontoblastic layer, as well as the predentin covering the mineralized dentine, must be exposed to initiate the process of resorption [7].

The predisposing factors to internal root resorption, as suggested in the literature include trauma, pulpitis, pulpotomy, cracked tooth, tooth transplantation, restorative procedures, invagination, orthodontic treatment, and even a Herpes zoster viral infection [3]. The clastic cells which are present in the apical zone of the resorptive lesion derive their nutrition from the blood cells which supply them with nutrients. On the other hand, necrotic-infected pulpal tissue stimulated the activity of clastic cells.

Sources of pathogenic microflora entering the pulpal space are via dentinal tubules due to the presence of cavities, microcracks, fractures, and a large number of lateral and accessory canals. Lack of these underlying exaggerating factors causes resorption to proceed in an otherwise sluggish and transient manner and might not be clinically but radiographically detectable. Thus, it is imperative to have a viable, healthy blood supply apical to the resorptive lesion to progress [3,8].

A variety of inflammatory and pro-inflammatory modulators are released from the accumulation of resorptive cells in the defect. These are primarily recruited to mediate the process of resorption to promote repair and hasten the underlying protective mechanisms. They are mainly cytokines that induce this process. Once the protective shield of the predentin, odontoblastic layer, and pre-cementum is disrupted, the bodily mechanisms of repair cannot take place, and the tooth becomes vulnerable [9,10]. Once begun, the course of resorption continues and is not stopped until the underlying cause is treated. Complete elimination of pulpal remnants halts the underlying resorptive process.

## Case presentation

A 28-year-old female patient resident of Yavatmal reported to the outpatient department with a chief complaint of discoloration and pus discharge in the upper front region involving the left lateral incisor tooth, of the jaw for seven days. The patient was apparently all right 10 years ago until she suffered a blow in the upper front region of the jaw, which was completely asymptomatic until seven days ago. However, the patient suffered another impact in the same region seven days ago that caused mild pain and the patient also started noticing discoloration tooth. The patient also noticed pus discharge, which appeared intermittently, sometimes causing mild pain. There was no history of heat or balm application, fever, swelling, night pain, or previous orthodontic treatment. The medical history of the patient was unremarkable; however, the patient had a habit of consuming areca nuts twice daily for three years.

On further questioning, she gave a history of a visit to a private dental clinic three days ago for an emergency root canal access opening in the same region. Extraoral inspection exhibited no gross facial asymmetry or swelling, which was noted. Intra-orally, the oral hygiene of the patient was satisfactory. On the pulp sensibility test, teeth 11 and 12 showed no response. However, both teeth were tender to percussion. Tooth 21 showed a delayed response in comparison to the contralateral and adjacent teeth, which showed equal responses.

**Figure 1:**
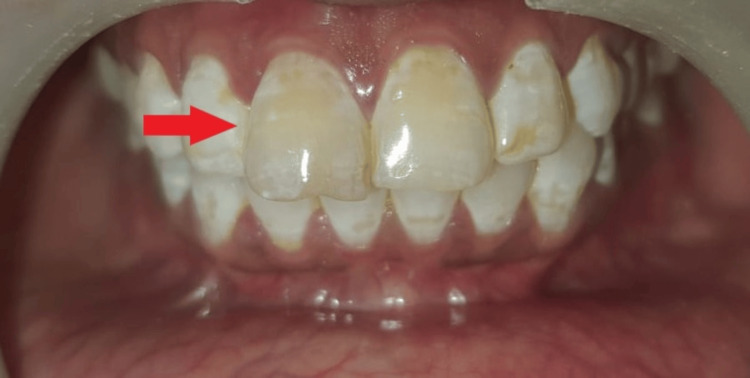
Clinical preoperative view showing discoloration and extrusion with tooth 11.

On intraoral examination, discoloration was seen and a slight amount of tooth extrusion was also present with tooth 11. An attempted previous root canal opening was seen in the palatal aspect of teeth 11 and 12 (Figure 1).

Radiographic assessment

**Figure 2:**
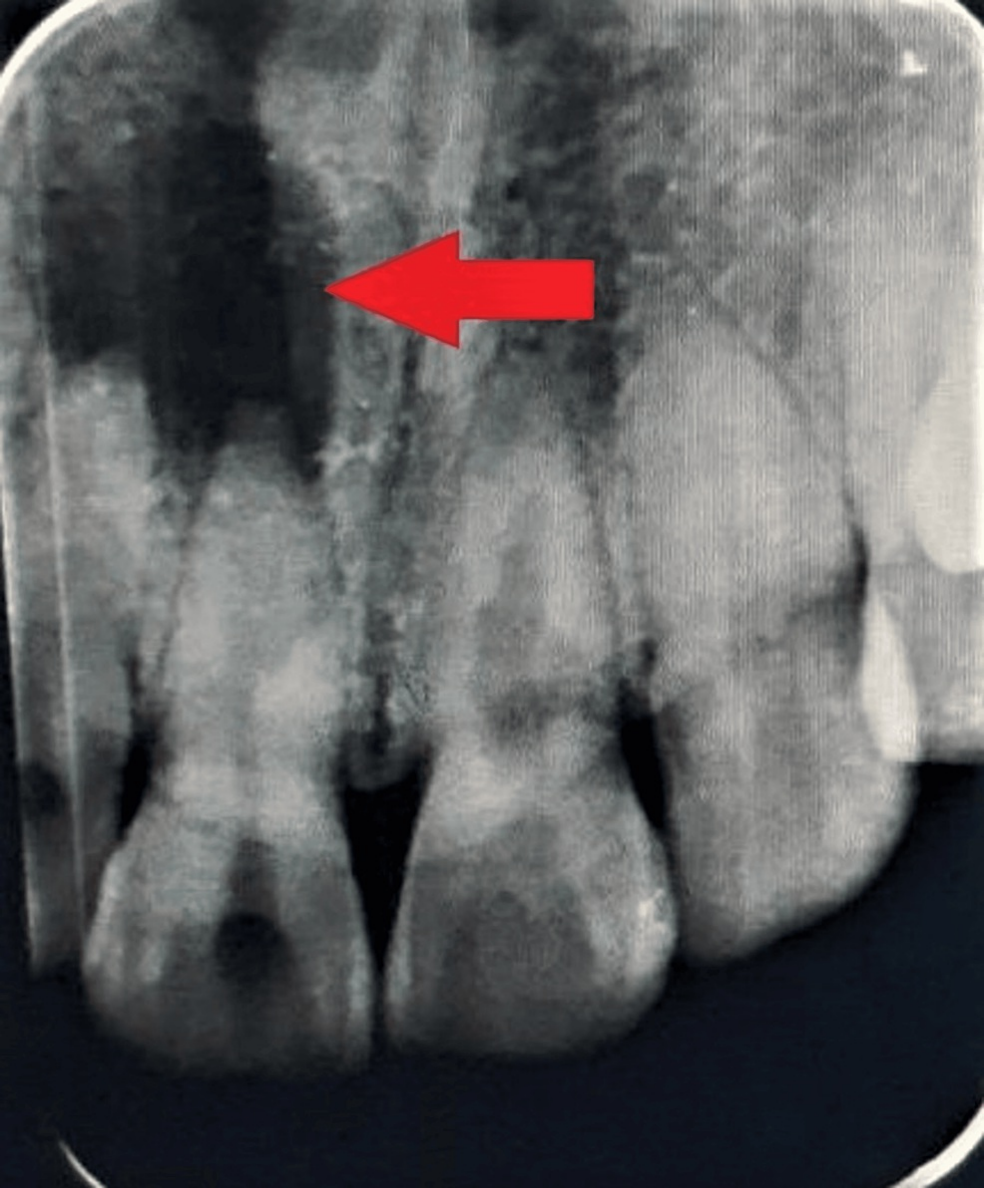
Single well-defined periapical radiolucency seen in the apex of teeth 11, 12.

In the intraoral periapical radiograph, a single large periapical radiolucent lesion is seen associated with 11, 12, which is suggestive of periapical abscess with 11, 12. An accidental finding showing a radiolucent defect with irregular boundaries was seen at the mid-root level of tooth 21 (Figure 2).

**Figure 3:**
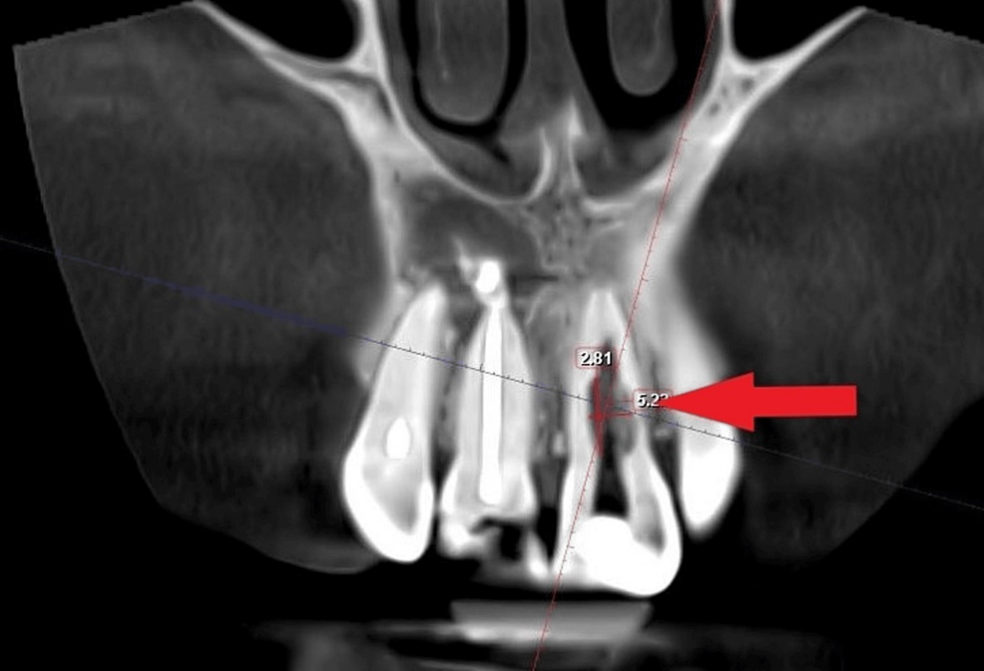
Coronal section of cone beam computed tomography showing the internal resorptive defect present in the mid-root region of tooth 21. The periapical abscess with teeth 11, 12 can also be appreciated.

Upon revelation of this accidental finding, the patient was further convinced to undergo a cone beam computed tomography (CBCT) investigation. CBCT revealed the size of the lesion in the coronal section, measuring 5.37 x 2.81 mm in length and breadth, respectively. The defect extended at the mid-root level of tooth 21 (Figure 3).

On the basis of diagnostic, clinical, and radiographic evidence since the lesion was non-perforating and non-communicating to the cementum, a final diagnosis of chronic periapical abscess with teeth 11 and 12 was given, while that of internal inflammatory root resorption was diagnosed with tooth 21.

Clinical procedure

**Figure 4:**
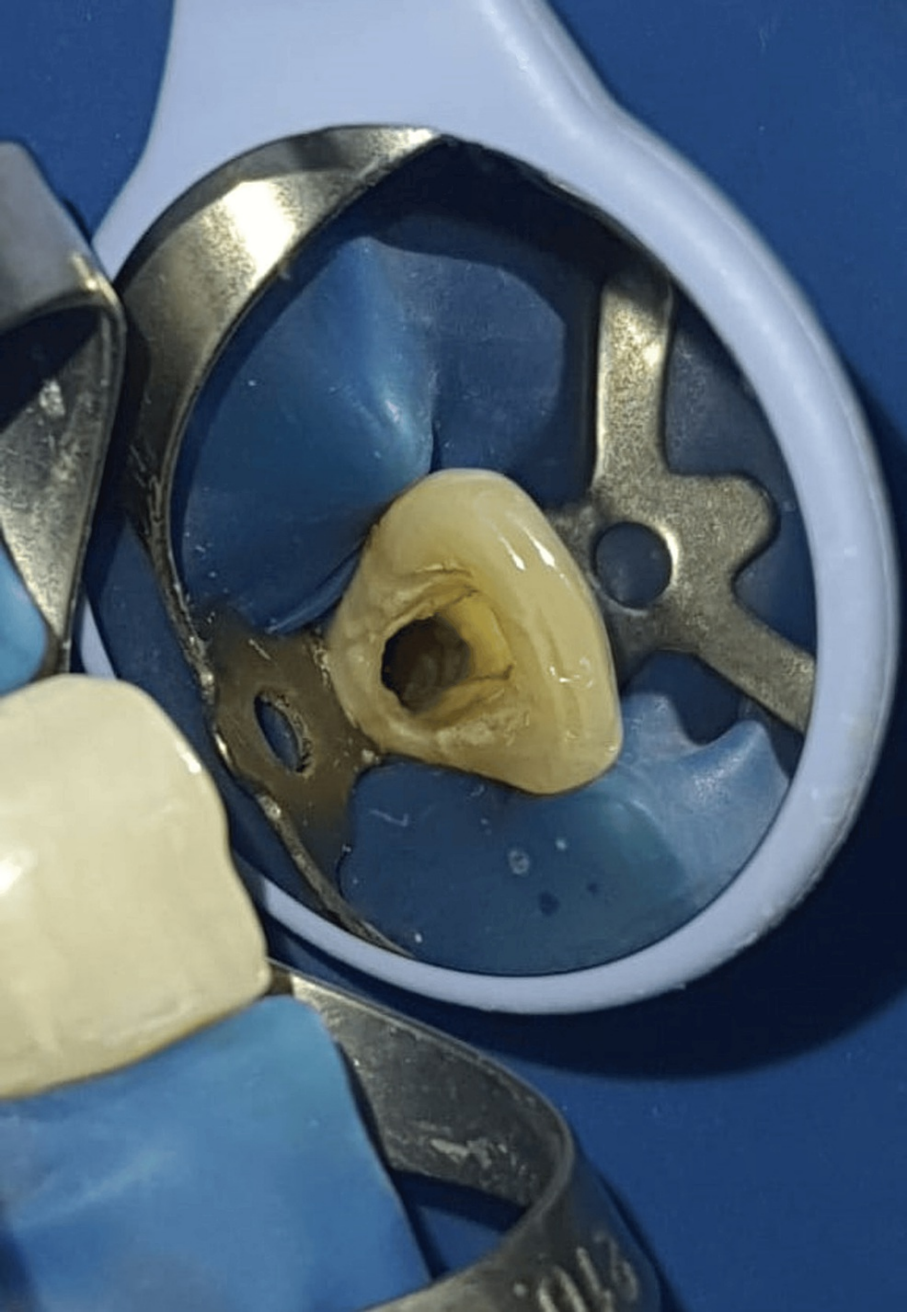
Access opening and biomechanical preparation done with tooth 21 under rubber dam isolation.

The patient was administered local anesthesia containing (1:100000) epinephrine (LOX 2%, Neon Laboratories, India). Under all aseptic conditions and proper rubber dam isolation, access opening was done with teeth 11 and 12 using a round end bur (BR-45 bur, Mani, Inc., Japan) and modified using a safe end bur (EX-24 bur, Mani, Inc., Japan). Working length was negotiated using an apex locator (JW Morita, Japan), and biomechanical preparation was done till 55 K file (Dentsply Sirona, United States) with 11, 21, and till 50 K file with 12. Further step-back preparation was done until the 70 K file with 11 and 21, followed by the 60 K file with 12. The working lengths were found to be 21 mm with both 11 and 21 and 20 mm with 12 (Figure 4).

**Figure 5:**
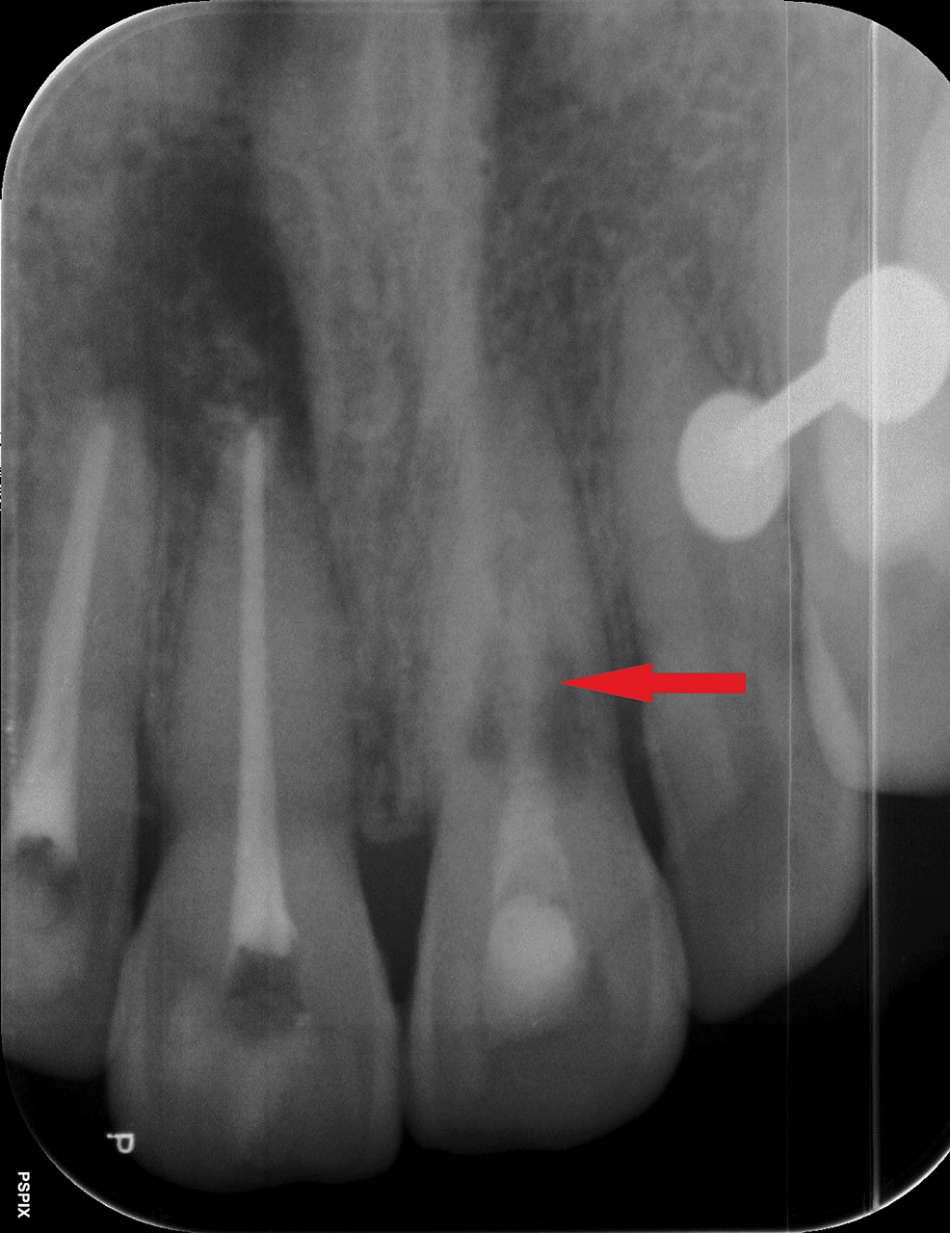
Obturation of teeth 11, 12 using gutta-percha. The arrow indicates the placement of intracanal medicament of calcium hydroxide in the resorptive defect of tooth 21.

This was followed by placing an intracanal dressing of calcium hydroxide in teeth 11 and 12 for a total duration of 15 days, with the medicament replaced after an interval of seven days. The patient was completely asymptomatic with respect to 11, 12 so it was decided to perform obturation followed by post-endodontic restoration with 11, 12 (Figure 5).

**Figure 6:**
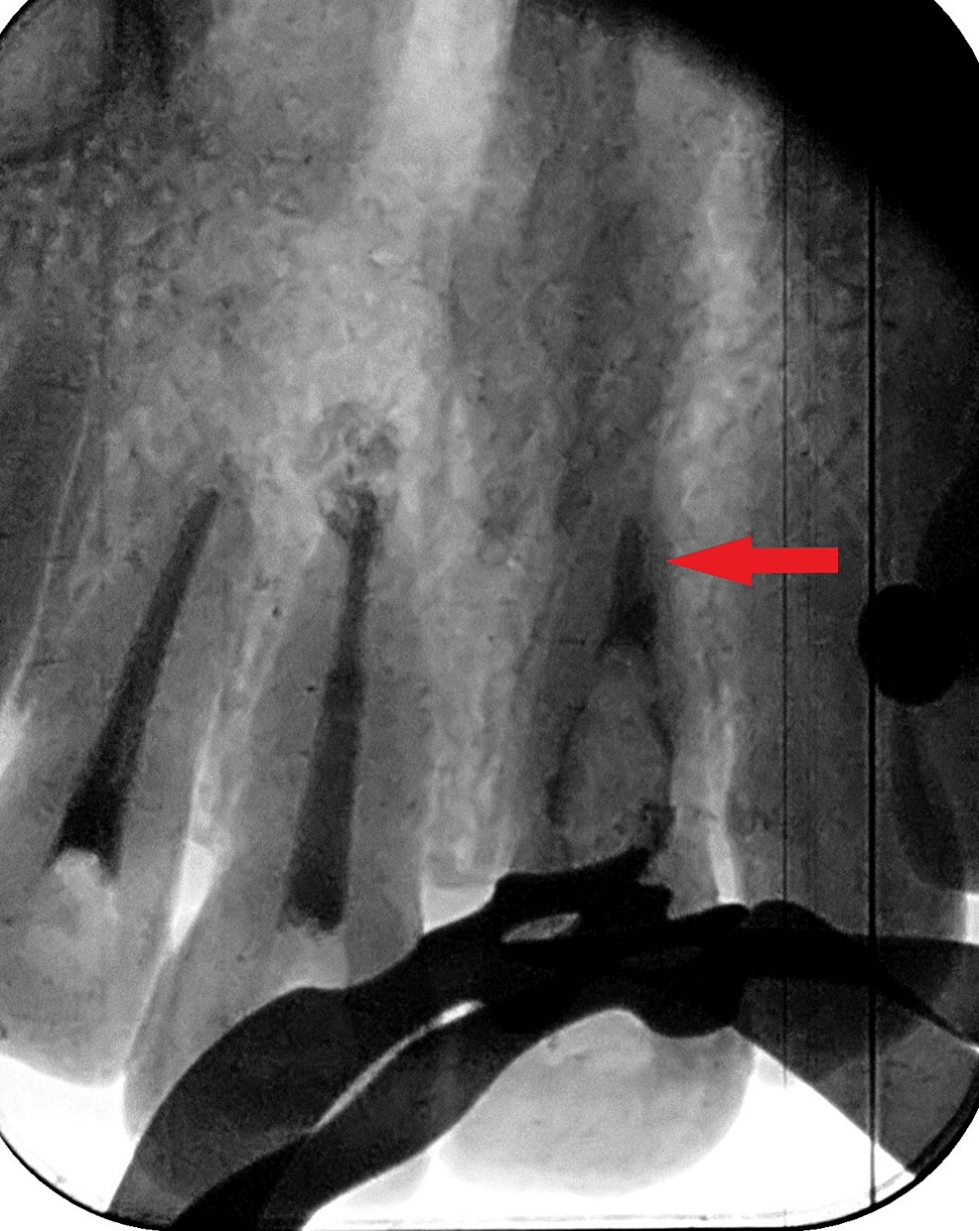
Contrast intraoral periapical radiograph with an arrow showing placement of mineral trioxide aggregate in the apical one-third of resorptive defect of tooth 21.

Intermittent, copious irrigation with 0.9% normal saline and 2% chlorhexidine was done before placing the medicament in the canal. For filling the resorptive defect in tooth 21, mineral trioxide aggregate (MTA) was chosen to be placed in the apical part and the remaining part of the resorptive defect was backfilled with thermo plasticized gutta-percha (System B, Sybron Endo, Kerr, Germany) (Figure 6).

**Figure 7:**
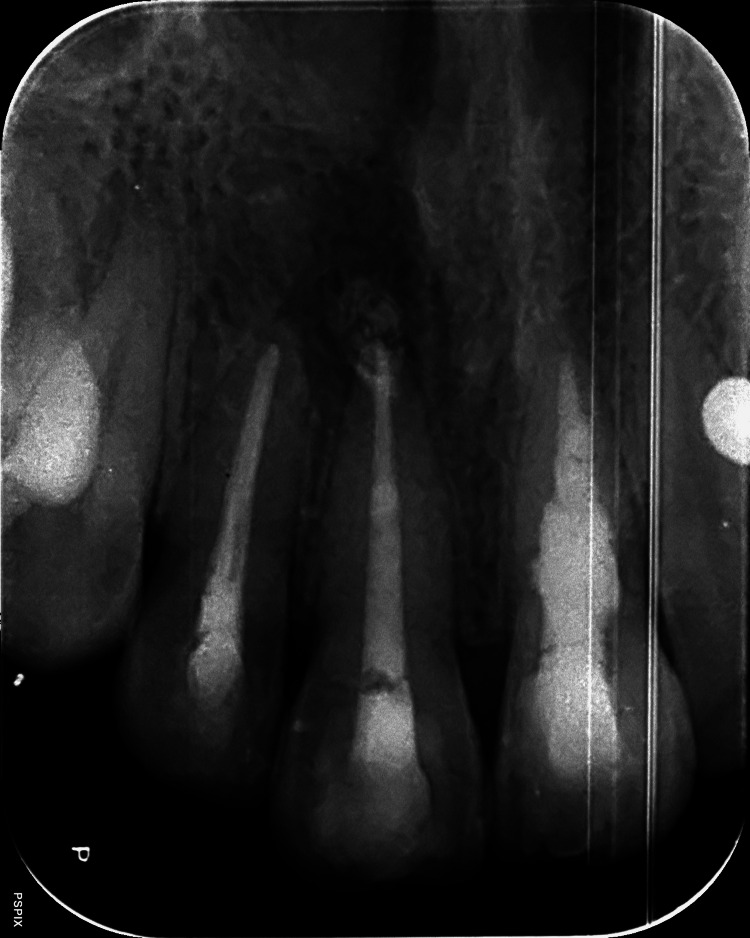
Intraoral periapical radiograph of follow-up after completion of treatment after six months showing significant periapical healing with all teeth.

The patient was evaluated periodically for follow-ups after one, three, and six months (Figure 7). 

## Discussion

Internal inflammatory root resorption, a deceptive process pathological in nature, commenced within the confines of the pulpal space, often linked to the degradation of dentin. The primary treatment of resorptive defects lies in treating the tooth by means of root canal treatment involving the usage of various bioactive materials. It is repeatedly termed an oval-shaped expansion of canal space and is frequently asymptomatic and evident only by means of advanced radiographs [11,12]. Frequently used materials for filling up the resorptive defects include gutta-percha, zinc oxide eugenol cement, and silver amalgam alloy. However, over time, these materials lose their potential to provide adequate strength to the tooth, do not provide strength to the tooth structure, and are responsible for causing discolorations to the tooth.

Intermittent dressings of calcium hydroxide aim to reduce the remaining pulpal debris present in the canal, creating an alkalizing environment and controlling periodontal bleeding. The use of white MTA was used for sealing the perforation as well as plugging the resorbed area. MTA was selected for its enhanced sealing properties as well as being a chosen and proven root repair material [13,14]. Clinical case scenarios where MTA experimented on humans demonstrated higher potentiality in moist environments, averting bacterial contamination and alkalinizing the canal medium. No significant evidence exists for the bonding of composites to other materials in MTA. This has not been reported before and warrants further investigation [15-17].

## Conclusions

The advent of proper radiographic imaging techniques makes the possibility of treating internal inflammatory root resorption easier and a less cumbersome procedure. The choice of availability of superior root canal filling materials provides the best possible outcomes to treat internal root resorptions. MTA and Biodentine serve as the best possible choice of materials to seal resorptive lesions. Bioceramic materials and bioactive glasses have been also tried to treat resorptions.

## References

[1] Patel S, Saberi N, Pimental T, Teng PH (2022). Present status and future directions: root resorption. Int Endod J.

[2] Gunraj MN (1999). Dental root resorption. Oral Surg Oral Med Oral Pathol Oral Radiol Endod.

[3] Patel S, Ricucci D, Durak C, Tay F (2010). Internal root resorption: a review. J Endod.

[4] Lyroudia KM, Dourou VI, Pantelidou OC, Labrianidis T, Pitas IK (2002). Internal root resorption studied by radiography, stereomicroscope, scanning electron microscope and computerized 3D reconstructive method. Dent Traumatol.

[5] Patel S (2009). New dimensions in endodontic imaging: part 2. Cone beam computed tomography. Int Endod J.

[6] de Paula-Silva FW, Wu MK, Leonardo MR, da Silva LA, Wesselink PR (2009). Accuracy of periapical radiography and cone-beam computed tomography scans in diagnosing apical periodontitis using histopathological findings as a gold standard. J Endod.

[7] Haapasalo M, Endal U (2006). Internal inflammatory root resorption: the unknown resorption of the tooth. Endod Top.

[8] Tronstad L (1988). Root resorption - etiology, terminology and clinical manifestations. Endod Dent Traumatol.

[9] Wedenberg C, Zetterqvist L (1987). Internal resorption in human teeth - a histological, scanning electron microscopic, and enzyme histochemical study. J Endod.

[10] Schaffner P, Dard MM (2003). Structure and function of RGD peptides involved in bone biology. Cell Mol Life Sci.

[11] Trope M (2002). Root resorption due to dental trauma. Endod Topics.

[12] Jacobovitz M, de Lima RK (2008). Treatment of inflammatory internal root resorption with mineral trioxide aggregate: a case report. Int Endod J.

[13] Arslan S, Balkaya H, Çakir NN (2019). Efficacy of different endodontic irrigation protocols on shear bond strength to coronal dentin. J Conserv Dent.

[14] Culbreath TE, Davis GM, West NM, Jackson A (2000). Treating internal resorption using a syringeable composite resin. J Am Dent Assoc.

[15] Torabinejad M, Hong CU, McDonald F, Pitt Ford TR (1995). Physical and chemical properties of a new root-end filling material. J Endod.

[16] Camilleri J, Pitt Ford TR (2006). Mineral trioxide aggregate: a review of the constituents and biological properties of the material. Int Endod J.

[17] Chandolu V, Mandava J, Borugadda R, Sirisha K, Kumar KR, Goteti S, Nallamilli LS (2024). Influence of access cavity design on root canal instrumentation efficacy in molars - an in vitro study. J Conserv Dent Endod.

